# The osmoregulated metabolism of trehalose contributes to production of type 1 fimbriae and bladder colonization by extraintestinal *Escherichia coli* strain BEN2908

**DOI:** 10.3389/fcimb.2024.1414188

**Published:** 2024-06-24

**Authors:** Vivian Souza Klemberg, Daniel Brisotto Pavanelo, Sébastien Houle, Sabin Dhakal, Pravil Pokharel, Simone Iahnig-Jacques, Charles M. Dozois, Fabiana Horn

**Affiliations:** ^1^ Departamento de Biofísica, Universidade Federal do Rio Grande do Sul, RS, Porto Alegre, Brazil; ^2^ Institut National de la Recherche Scientifique (INRS)-Centre Armand-Frappier Santé Biotechnologie, Laval, QC, Canada

**Keywords:** ExPEC, extraintestinal *E. coli*, BEN2908, trehalose metabolism, type 1 fimbriae

## Abstract

In *Escherichia coli*, the disaccharide trehalose can be metabolized as a carbon source or be accumulated as an osmoprotectant under osmotic stress. In hypertonic environments, *E. coli* accumulates trehalose in the cell by synthesis from glucose mediated by the cytosolic enzymes OtsA and OtsB. Trehalose in the periplasm can be hydrolyzed into glucose by the periplasmic trehalase TreA. We have previously shown that a *treA* mutant of extraintestinal *E. coli* strain BEN2908 displayed increased resistance to osmotic stress by 0.6 M urea, and reduced production of type 1 fimbriae, reduced invasion of avian fibroblasts, and decreased bladder colonization in a murine model of urinary tract infection. Since loss of TreA likely results in higher periplasmic trehalose concentrations, we wondered if deletion of *otsA* and *otsB* genes, which would lead to decreased internal trehalose concentrations, would reduce resistance to stress by 0.6 M urea and promote type 1 fimbriae production. The BEN2908Δ*otsBA* mutant was sensitive to osmotic stress by urea, but displayed an even more pronounced reduction in production of type 1 fimbriae, with the consequent reduction in adhesion/invasion of avian fibroblasts and reduced bladder colonization in the murine urinary tract. The BEN2908Δ*treAotsBA* mutant also showed a reduction in production of type 1 fimbriae, but in contrast to the Δ*otsBA* mutant, resisted better than the wild type in the presence of urea. We hypothesize that, in BEN2908, resistance to stress by urea would depend on the levels of periplasmic trehalose, but type 1 fimbriae production would be influenced by the levels of cytosolic trehalose.

## 1 Introduction

Trehalose is an α(1➔1)-dimer of glucose and is present in organisms of all Domains of life, being absent only in vertebrates (Germer et al., 1998; Joseph et al., 2010; Argüelles, 2014). In all organisms that have been studied, trehalose plays a dual role: as an energy source and as a protector against various forms of stress, such as high osmolarity, dehydration, heat, cold and oxidative stress (Elbein et al., 2003; Ruhal et al., 2013). Accordingly, *Escherichia coli* can metabolize trehalose as a carbon source, or can accumulate it under osmotic stress or low temperature (Boos et al., 1990; Kandror et al., 2002). Under isotonic conditions, *E. coli* K-12 internalizes trehalose through a phosphotransferase system (EIICB^Tre^, encoded by *treB*) (Rimmele and Boos, 1994), and trehalose-6-phosphate is then hydrolyzed by cytosolic trehalose-6-phosphate hydrolase (encoded by *treC*) into two glucose molecules, which are used in glycolysis (Rimmele and Boos, 1994). Under hypertonic conditions, however, trehalose is synthesized internally from glucose-6-phosphate and UDP-glucose in two reactions, catalyzed by the osmoregulated-trehalose synthesis (Ots) enzymes trehalose-6-phosphate synthase (encoded by *otsA*) and trehalose-6-phosphate phosphatase (encoded by *otsB*) (Giaever et al., 1988). Any trehalose sent to the periplasm is degraded into two glucose molecules by the periplasmic trehalase (encoded by *treA*), whose activity is also increased under osmotic stress (Boos et al., 1987). The glucose, in turn, is transported back to the cytoplasm by the glucose phosphotransferase system (EIICB^Glu^) to be a substrate for cytoplasmic trehalose synthesis (Strom and Kaasen, 1993; Li et al., 2012).

In recent years, a relationship between trehalose metabolism and virulence has been demonstrated in a few bacterial pathogens. Collins et al. (2018) reported that *Closteroides difficile* ribotypes RT027 and RT078 emerged as major epidemic ribotypes shortly after trehalose had been introduced as an additive in industrialized food. The hypothesis raised was that these *C. difficile* ribotypes emerged as a result of their ability to metabolize low concentrations of trehalose, conferring a competitive advantage over the intestinal microbiota. In *Ralstonia solanacearum*, biosynthesis of trehalose via the OtsA and OtsB enzymes was proposed to play a fundamental role in successful plant colonization and infection, thus being one of the virulence/fitness mechanisms that contributes to tomato wilt disease (MacIntyre et al., 2020). In *Burkholderia pseudomallei*, loss of *treA* increased tolerance to thermal stress but reduced biofilm production and bacterial survival in macrophages (Vanaporn et al., 2017).

In a previous report (Pavanelo et al., 2018), we determined that trehalose metabolism influences virulence of an extraintesinal pathogenic *Escherichia coli* (ExPEC) strain of avian origin BEN2908 (previously known as MT78). In a screening of BEN2908’s transposon (STM) mutants, we reported that a *treA* null mutant was reduced in production of type 1 fimbriae, demonstrated decreased adherence and invasion of avian fibroblasts and decreased bladder colonization in the murine model of urinary tract infection (UTI) (Pavanelo et al., 2018). BEN2908Δ*treA* also showed increased resistance to osmotic stress caused by urea (but not by NaCl), presumably caused by increased levels of trehalose in the periplasm due to the lack of its periplasmic trehalase (Pavanelo et al., 2018).

In this work, we investigated whether reduced levels of type 1 fimbriae in BEN2908Δ*treA* could potentially be due to higher levels of periplasmic trehalose that could have accumulated in the absence of the periplasmic trehalase, TreA. To answer this, we generated a mutant lacking the *otsA* and *otsB* genes required for trehalose synthesis (Δ*otsBA*) as well as a mutant lacking *otsA* and *otsB* and the *treA* gene required for hydrolysis of periplasmic trehalose (Δ*treAotsBA*), and analyzed their phenotypes for bacterial growth in the presence of NaCl and urea, production of type 1 fimbriae determined by yeast agglutination, and adhesion and invasion of CEC-32 avian fibroblasts. The trehalose synthesis mutant was also tested in the murine UTI model.

## 2 Results

### 2.1 Loss of trehalose synthesis decreased growth of BEN2908 on 0.6 M urea

**Figure 1:**
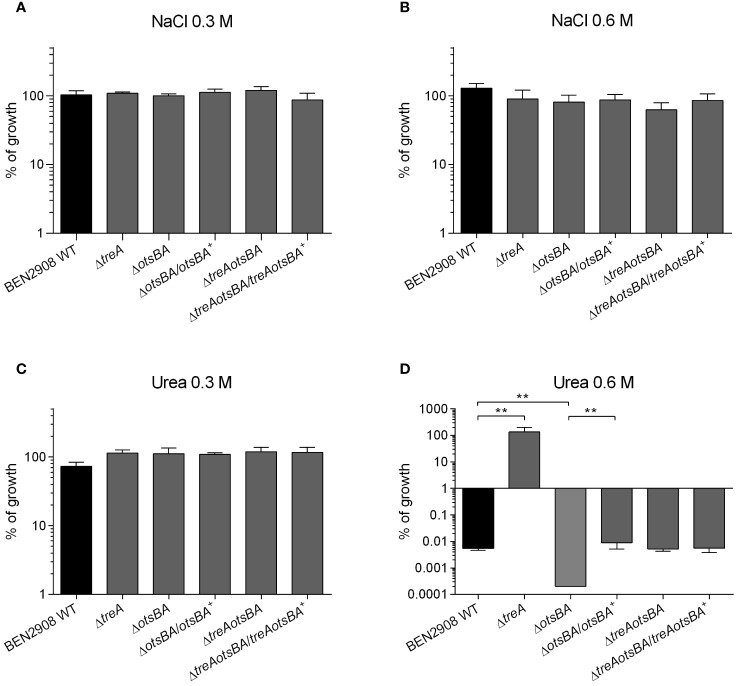
Growth of BEN2908 WT and mutants on LB agar under conditions of osmotic stress. Strains were grown under shaking in LB medium until mid-log phase (OD_600_ = 0.6) and plated on LB agar (without added NaCl; taken as 100% growth) and on LB agar with 0.3 M of NaCl **(A)**, 0.6 M of NaCl **(B)**, 0.3 M of urea **(C)**, and 0.6 M of urea **(D)**. The Δ*otsBA* mutant showed no growth on plates with 0.6 M urea (0 CFU), % growth were given a % growth value of 0.0002%. The values are the average growth relative to CFUs on LB agar (without NaCl) ± SEM of three and four experiments (biological replicates) performed in duplicates (technical replicates) for NaCl and urea, respectively. **, *P* < 0.01 (Mann-Whitney test).

In *E. coli* intracellular production of trehalose as an osmoprotectant has been characterized mainly in response to external osmotic strength caused by NaCl. However, the Δ*treA* mutant of BEN2908, unable to hydrolyze trehalose in the periplasm, did not show increased osmoprotection against NaCl, but did so against urea (Pavanelo et al., 2018). To verify how the BEN2908 Δ*otsBA* and Δ*treAotsBA* mutants, unable to synthesize trehalose, respond to stress caused by these two molecules, mutants and complemented strains were grown on LB agar containing NaCl or urea, and were compared to growth of the WT and Δ*treA* mutant. In the presence of 0.3 M and 0.6 M NaCl or 0.3 M urea (average concentration of urea in human urine) all strains displayed similar growth (**Figures 1A–C**). However, in the presence of 0.6 M urea, where the growth of the WT was severely impaired and the Δ*treA* mutant grew as well as in the absence of urea (as previously reported (Pavanelo et al., 2018), the Δ*otsBA* was unable to grow (**Figure 1D**). Trans-complementation of the Δ*otsBA* mutant restored growth to WT levels on medium containing 0.6 M urea. Growth of the Δ*treAotsBA* mutant, lacking all the osmoregulated enzymes of trehalose metabolism, also grew similarly to the WT in the presence of 0.6 M urea.

### 2.2 OtsA, OtsB, and TreA are not required for growth with trehalose as the only carbon source

At low osmolarity, import and catabolism of trehalose by the TreB/TreC pathway are induced in the presence of trehalose in the growth medium, whereas under osmotic stress *E. coli* accumulates trehalose internally through the osmoregulated OtsA/OtsB/TreA pathway (Strom and Kaasen, 1993). Since the common molecule between these pathways is trehalose-6-phosphate (also an inducer of the trehalose import system) (Klein et al., 1991), we wondered if the absence of the OtsA/OtsB/TreA pathway is required for growth when trehalose is the sole carbon source.

**Figure 2:**
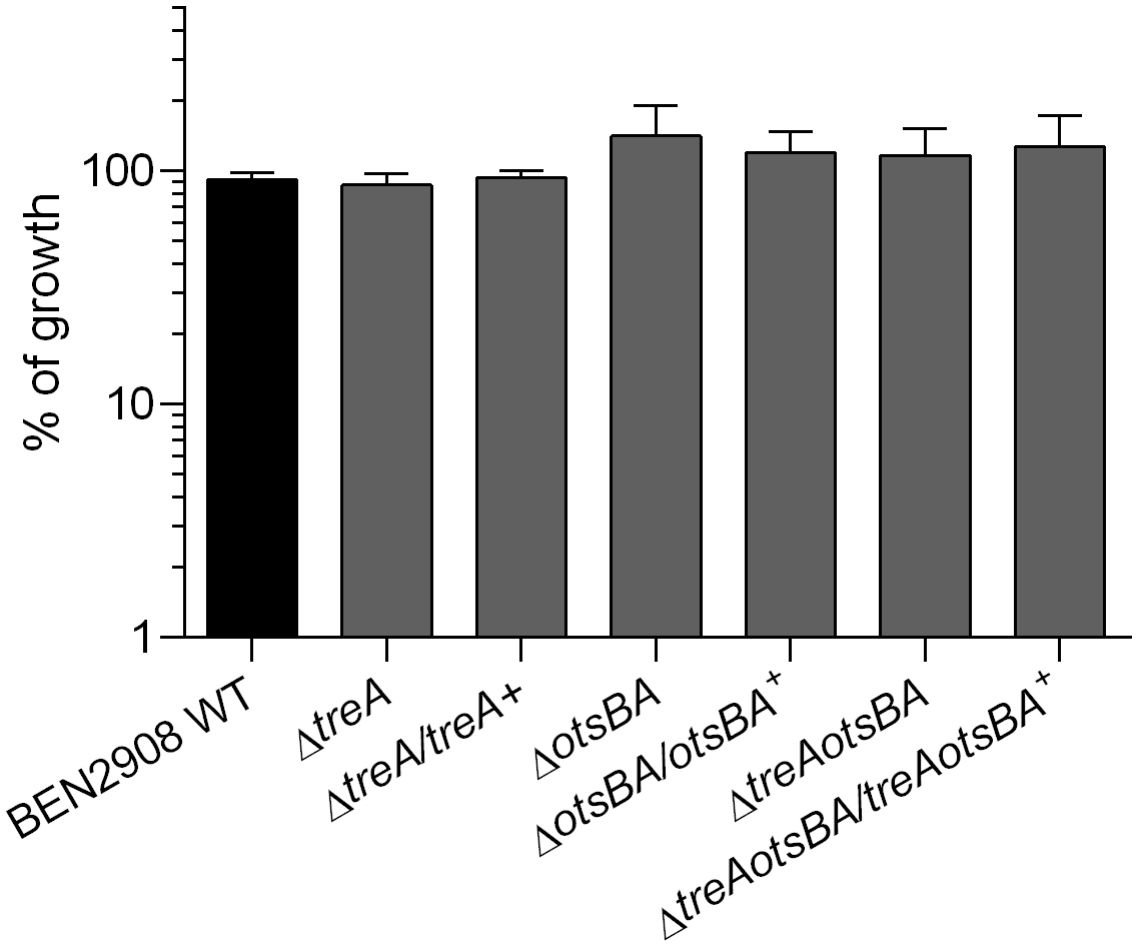
Growth of BEN2908 WT and mutants on M9 agar supplemented with 10 mM trehalose as the sole carbon source. Strains were grown in LB medium until mid-log phase (OD_600_ = 0.6) and plated on M9 agar with glycerol 0.6% (taken as 100% growth) or with trehalose 10 mM as the sole carbon source. The values are the average growth relative to M9 agar with glycerol 0.6% ± SEM of three experiments performed in duplicates.

We have previously not observed growth of the Δ*treA* mutant in M9 minimal medium with trehalose as the carbon source at 24 h (Pavanelo et al., 2018). Now we tested growth of the Δ*treA*, Δ*otsBA* and Δ*treAotsBA* mutants on M9-agar supplemented with 0.6% glycerol or 10 mM trehalose as the only carbon source. All mutants grew as well as the WT with either glycerol or trehalose as the sole carbon source (**Figure 2**), although at 24 h colonies were too small and were only visible after 48 h of incubation. It is thus unlikely that the absence of the OtsA/OtsB/TreA pathway impairs trehalose assimilation by the TreB/TreC pathway. The slow growth may explain why we did not previously observe growth of the Δ*treA* mutant in M9 minimal medium with trehalose as the sole carbon source after 24 h (Pavanelo et al., 2018).

### 2.3 Loss of *treA* or *otsBA* reduced yeast agglutination titer

**Figure 3:**
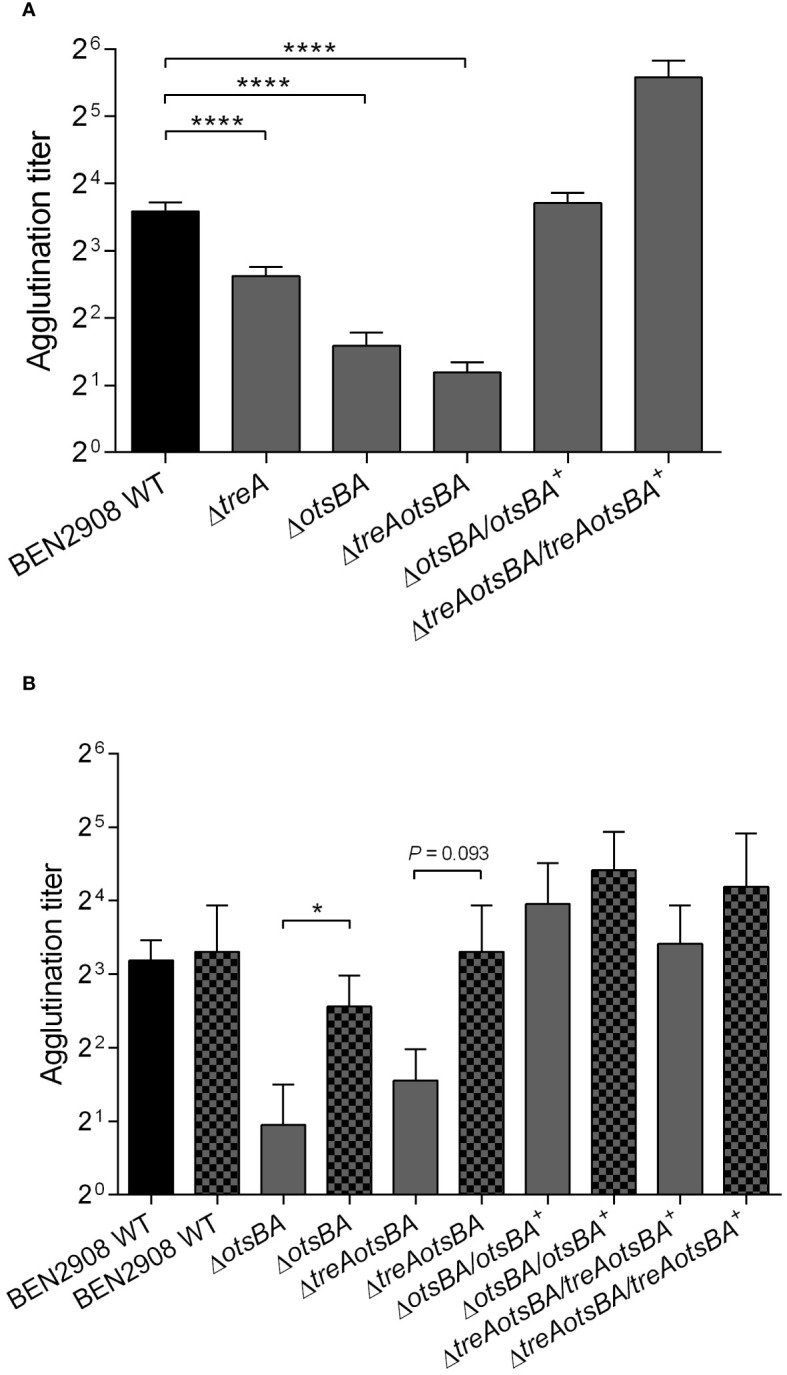
Yeast agglutination of BEN2908 WT and mutants was measured after mid-log growth (OD_600nm_= 0.6) with shaking in LB broth **(A)** and in LB broth without NaCl **(B)** in the absence (left bar of each pair) and presence of 0.3 M urea (right, patterned bar of each pair),. The values are the average ± SEM of eleven and three experiments done in duplicates for A and B, respectively. ****P < 0.0001 (Mann-Whitney test) in **(A)**; * *P* < 0.05 (Unpaired t test) in **(B)**.

Because the observed decrease in type 1 fimbriae production in BEN2908Δ*treA* may result from increased levels of periplasmic trehalose (as suggested by greater resistance to stress by urea in this mutant) (Pavanelo et al., 2018), we wondered whether suppression of trehalose synthesis would have the opposite effect, i.e., would possibly increase type 1 fimbriae production by strain BEN2908. We performed yeast agglutination assays with the WT, and the Δ*treA*, Δ*otsBA* and Δ*treAotsBA* mutants, as well as their respective complemented strains. **Figure 3A** shows that, while the Δ*treA* mutant had a two-fold reduction in the production of type 1 fimbriae, the Δ*otsBA* and Δ*treAotsBA* mutants had a four-fold reduction when compared to the WT parent strain. Further, trans-complementation of Δ*otsBA* mutant restored yeast agglutination titers to WT levels, or higher for complementation of the Δ*treAotsBA* mutant. These results suggest that changes in osmoregulation due to altered trehalose metabolism either through loss of trehalose *de novo* synthesis or lack of degradation in the periplasm may contribute to a decrease in type 1 fimbriae production.

### 2.4 Growth with 0.3 M urea increased type 1 fimbriae production by the Δ*otsBA* mutant

The absence of the enzymes for *de novo* synthesis of trehalose (*otsA* and *otsB*) rendered strain BEN2908 more sensitive to 0.6 M urea (**Figure 1D**), indicating that synthesis of trehalose provided osmoprotection against urea, in line with our previous observations (Pavanelo et al., 2018). On the other hand, 0.6 M urea upregulated expression of *fim* genes in the UPEC strain CFT073 (Withman et al., 2013). Since optimal production of type 1 fimbriae required the osmoregulated synthetic enzymes of trehalose (**Figure 3A**), we wondered if urea could trigger type 1 fimbriae production through increased synthesis of trehalose by OtsA and OtsB.

To verify this hypothesis, strains were grown in 0.3 M urea and tested for production of type 1 fimbriae by yeast agglutination (**Figure 3B**). The *otsAB* mutant displayed a significantly higher agglutination titer when grown in the presence of 0.3 M urea, indicating that urea-mediated upregulation of type 1 fimbriae production is independent of metabolism of trehalose.

### 2.5 Swimming motility of WT and mutants

**Figure 4:**
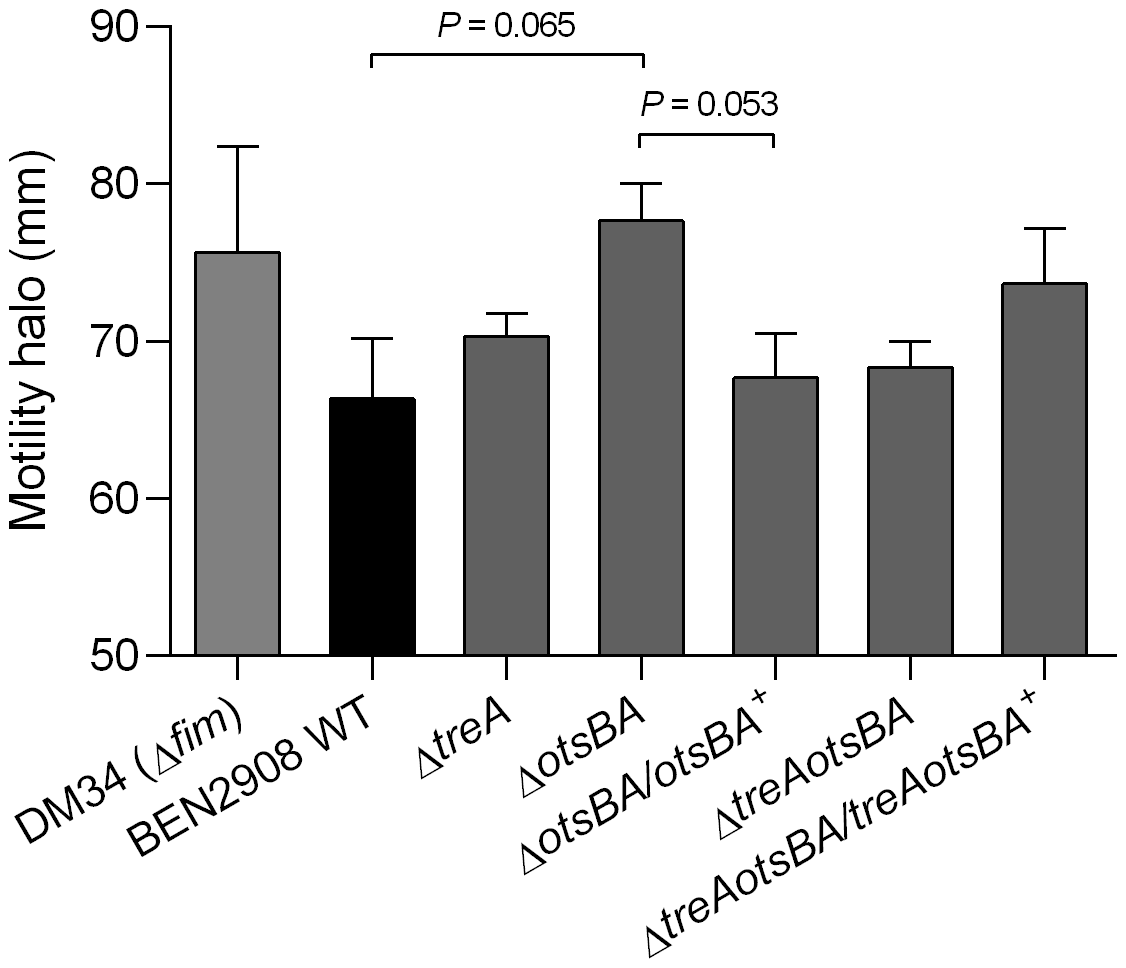
Diameters of swimming motility of BEN2908, mutants and complemented mutants. DM34 strain, a *fim* null mutant of BEN2908, was used as a positive control. The swimming motility was verified on soft agar (1% tryptone, 0.5% NaCl, 0.3% agar). Strains were stabbed onto the surface of soft agar and incubated at 37° C for 16 hours. The values represent the average ± SEM of three experiments done in duplicates. Significant differences in motility were analyzed using unpaired t-test.

A decrease in type 1 fimbriae expression in *E. coli* may also result in an increase in flagella-mediated motility (Lane et al., 2007; Spurbeck and Mobley, 2013). To determine if this occurs in BEN2908, we tested the swimming motility of WT and mutant strains (**Figure 4**). Despite the absence of statistical significance, there was an observed increase in motility for the *otsAB* mutant, to levels similar to the *fim* null mutant DM34. The complemented strain rescued the WT phenotype.

### 2.6 Adherence to avian fibroblasts was decreased for *ΔotsBA* and *ΔtreAotsBA* mutants

**Figure 5:**
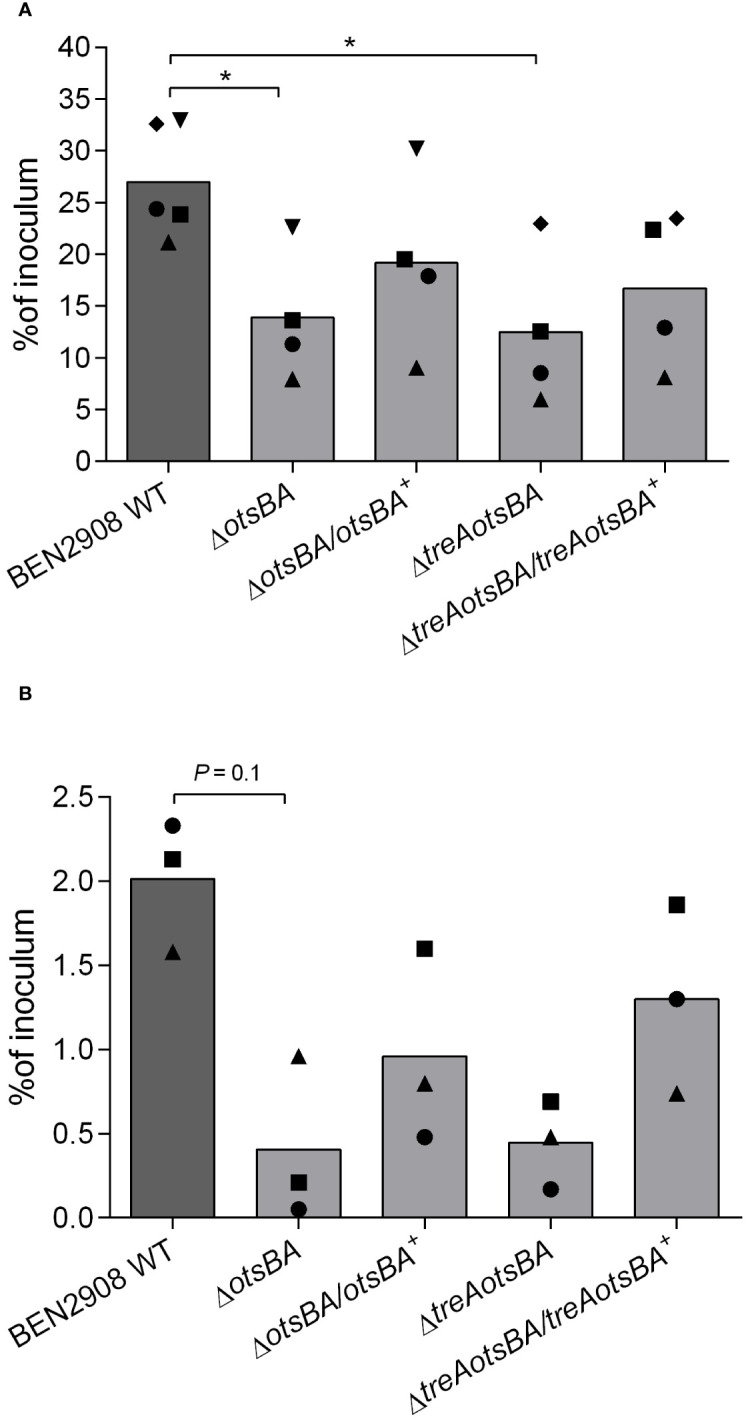
Adhesion to and invasion of CEC-32 avian fibroblasts. Cells were infected at an MOI of 20 CFU/cell. Bars represent the median of recovered CFU compared to that of inoculum 1 h after infection (adhesion) **(A)** and 4 h after infection, the first hour without gentamicin, followed by washes and reincubation in the presence of gentamicin at 50 µg/mL for a further 3 h (invasion) **(B)**. Data are from four and three experiments done in triplicates, respectively. Same symbols represent data from the same experiment. *P < 0.05 (Mann-Whitney test).

*E. coli* type 1 fimbriae contribute to virulence and play a crucial role in bacterial adhesion (Kaper et al., 2004; Mobley et al., 2013). Since adhesion to and invasion of CEC-32 fibroblasts are dependent on type 1 fimbriae production, we wondered if BEN2908Δ*otsBA* and BEN2908Δ*treAotsBA* mutants, that produce less type 1 fimbriae than WT, would be impaired in their adherent and invasive capacities. While 27% of the initial inoculum of the WT strain adhered to avian fibroblasts after 1 hour of interaction, the Δ*otsBA* and Δ*treAotsBA* mutants adhered significantly less (*P* < 0.05) with respectively only 14% and 13% adherence of the initial inoculum (**Figure 5A**). There was also a reduction in the invasion levels of the mutants compared to WT, although this was not statistically significant (**Figure 5B**).

### 2.7 The *ΔotsBA* mutant was less able to colonize the bladder during murine urinary tract infection

**Figure 6:**
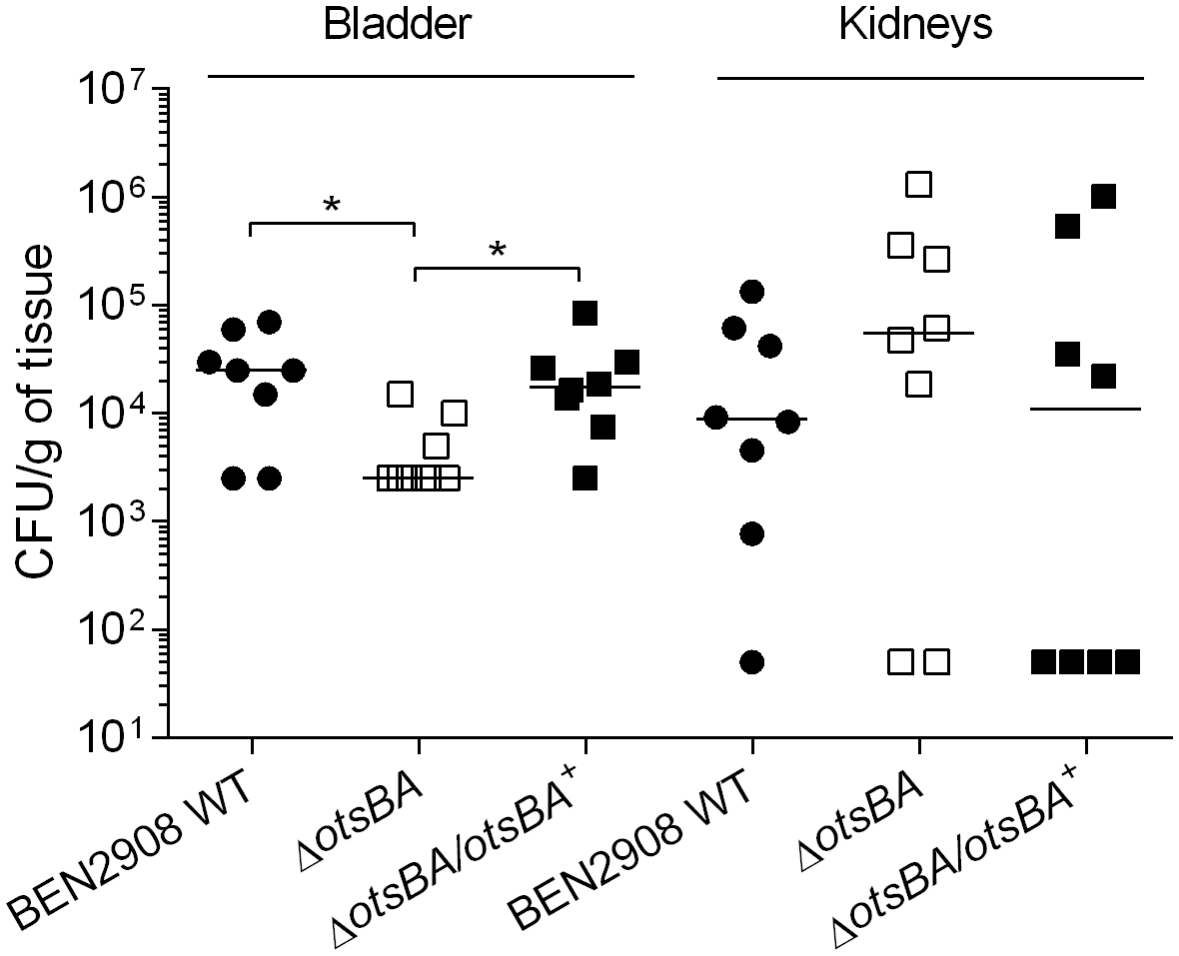
Loss of trehalose synthesis reduces bladder colonization during urinary tract infection in mice. CBJ/A mice were infected transurethrally, the animals were euthanized, and bladder and kidneys were collected 48 h post-infection. For bladders, each data point represents bacterial numbers recovered from the bladder of an individual mouse. For kidneys, each data point represents the mean of both right and left kidneys from the same mouse. Horizontal bars indicate the medians. *P < 0.05 (Mann-Whitney test).

Type 1 fimbriae are important for adherence and colonization of *E. coli* in the urinary tract. BEN2908 WT strain is able to cause urinary tract infection in a murine model, and BEN2908Δ*treA* presents a reduced ability to colonize the murine bladder (Pavanelo et al., 2018). We therefore tested whether the decrease in type 1 fimbriae production observed *in vitro* would also reduce bladder or kidney colonization in a murine model of urinary tract infection. Indeed, the *otsBA* mutant colonized the murine bladder 10-fold less than the WT (*P* < 0.05), whereas the complemented mutant colonized the bladder at levels similar to the WT (**Figure 6**). There was no significant difference in colonization of the kidney between the mutant and the WT strain.

## 3 Discussion

To resist osmotic stress, bacteria produce osmolytes to counteract the high external osmolarity and avoid fluid loss, thus maintaining the turgor by reducing water activity (Ruhal et al., 2013). In *E. coli*, the osmoprotectant trehalose has been shown to accumulate in cells in response to osmotic stress by monovalent cations (Strom and Kaasen, 1993). The increased levels of intracellular trehalose in response to NaCl are a consequence of increased activities of the cytoplasmic synthetic enzymes OtsA and OtsB (Boos et al., 1987; Giaever et al., 1988) and of the periplasmic trehalase, TreA (Boos et al., 1990). A 2-fold increase in expression of *otsA* and *otsB* in the presence of NaCl (but not in urea) has been observed in uropathogenic *E. coli* strain CFT073 (Withman et al., 2013). *E. coli* mutants unable to synthesize trehalose have impaired growth under osmotic stress caused by NaCl (Giaever et al., 1988). Contrary to what has been observed for *E. coli* K-12, deletion of the osmoregulated enzymes of trehalose metabolism had no effect on growth of avian pathogenic *E. coli* strain BEN2908 when osmolarity was increased with NaCl (**Figure 1A, B**).

We have previously shown that deletion of *treA* in strain BEN2908 resulted, on one hand, in increased resistance to osmotic stress caused by the permeant osmolyte urea, and, on the other hand, in decreased production of type 1 fimbriae. This strain consequently was less able to adhere to and invade avian fibroblasts and had decreased bladder colonization in the murine urinary tract (Pavanelo et al., 2018). As the Δ*treA* mutant most likely accumulates trehalose in the periplasm, we wondered whether decreasing the endogenous levels of trehalose by deleting the cytosolic enzymes of trehalose synthesis would potentially cause reduced osmotic resistance and enhanced type 1 fimbriae production. In contrast to some other bacterial species such as *Pseudomonas* or *Ralstonia* (Freeman et al., 2010; Djonović et al., 2013; MacIntyre et al., 2020), synthesis of trehalose in *E. coli* is completely dependent on OtsA and OtsB enzymes, and deletion of *otsAB* abrogates trehalose synthesis (Giaever et al., 1988).

We first tested the resistance of WT and null mutants to osmotic stress induced by urea. While the Δ*treA* mutant grew better than the WT parent in minimal medium in the presence of 0.6 M urea, in agreement with our previous results (Pavanelo et al., 2018), Δ*otsBA*, a mutant that is unable to synthesize trehalose, grew less than the WT under the same conditions (**Figure 1D**). The growth defect of the Δ*otsBA* mutant in urea was alleviated by additional loss of *treA* encoding the periplasmic trehalase TreA (Δ*treAotsBA*, **Figure 1D**). This result suggests that loss of all osmoregulated enzymes involved in trehalose metabolism likely results in increased production of other osmoprotectants, such as betaines, in response to osmotic stress caused by urea (Strom and Kaasen, 1993; Randall et al., 1996).

In *E. coli*, periplasmic trehalose can have two distinct fates. Under hypertonic conditions, trehalose is hydrolyzed by TreA, and the resulting glucose molecules can be internalized and serve as the precursors for cytoplasmic trehalose synthesis (by OtsA and OtsB). Under isotonic conditions, trehalose is transported to the cytoplasm via a trehalose-specific PTS system (TreB); trehalose-6-phosphate can then be hydrolyzed by the cytosolic trehalose-6-phosphate hydrolase (TreC) to glucose and glucose-6-P, which can be substrates in the glycolysis pathway. BEN2908Δ*otsBA*, Δ*treAotsBA* and *ΔtreA* mutants were able to grow on minimal medium with trehalose as the only carbon source (**Figure 2**), indicating that the genes responsible for metabolism of trehalose in high osmotic conditions were not required for utilization of trehalose as a carbon source when *E. coli* is grown under low osmotic conditions, as reported previously (Boos et al., 1990).

**Figure 7:**
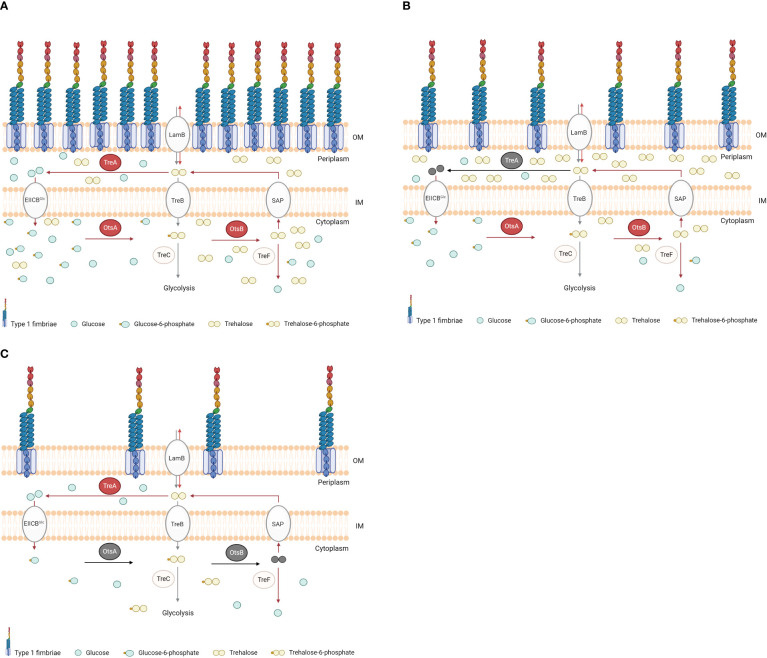
Diagram illustrating our hypothesis for the periplasmic and cytosolic concentrations of trehalose in BEN2908 wild type and in Δ*treA* and Δ*otsBA* mutants. The respective phenotypes of type 1 fimbriae are shown. In WT (**A**), the osmoregulated-trehalose synthesis (Ots) enzymes trehalose-6-phosphate synthase (OtsA) and trehalose-6-phosphate phosphatase (OtsB), and the periplasmic trehalase TreA, whose activities are increased under osmotic stress (as indicated by arrows in red), keep basal concentrations of trehalose in cytosol and periplasm. In the Δ*treA* mutant (**B**, deletion indicated in grey), absence of trehalose hydrolysis results in increased concentrations of periplasmic trehalose, thus protecting against extracellular urea, while lowering the quantity of glucose available to enter the cytosol (through EIICB^Glu^) and serve as substrate for OtsA-OtsB to synthesize cytosolic, endogenous trehalose. As consequence, levels of cytosolic trehalose are reduced in the Δ*treA* mutant. In the Δ*otsBA* mutant (**C**, deletions indicated in grey), absence of trehalose synthesis in the cytosol lowers trehalose concentrations both in cytosol and periplasm, rendering the mutant more susceptible than WT to urea. Any trehalose in the periplasm would be of exogenous source. The defect in type 1 fimbriae production observed in the Δ*treA* and Δ*otsBA* mutants could be explained by lower levels of cytosolic trehalose, through an as yet unknown mechanism. OM, outer membrane; IM, inner membrane; LamB, sugar transporter; EIIA^Glc^, glucose phosphotransferase system; TreB, trehalose-specific PTS system; SAP, stretch-activated protein; TreC, trehalose-6-phosphate hydrolase; TreF, cytoplasmic trehalase.

The *treA* mutant of BEN2908 produced less type 1 fimbriae, which likely resulted in reduced invasion of avian fibroblasts and reduced bladder colonization of the murine urinary tract (Pavanelo et al., 2018). In urinary tract infections, bladder colonization by uropathogenic *E. coli* (UPEC) depends on the binding of type 1 fimbriae to host cell uroplakin, which can promote bacterial internalization (Mulvey et al., 1998). Without type 1 fimbriae, *E. coli* is less able to establish urinary tract colonization. Notably, *fimA* (encoding the major subunit of type 1 fimbriae) is the fourth most expressed protein in UPEC strain CTF073 following infection in mice, only behind three proteins involved in protein synthesis machinery (Snyder et al., 2004). Moreover, when strain CFT073 was grown in the presence of 0.6 M urea (but not when supplemented with 0.3 M NaCl), expression of *fim* genes was upregulated ~4-fold (Withman et al., 2013). Neonatal meningitis-causing *E. coli* is thought to require type 1 fimbriae to adhere to human brain microendothelial cells and may promote traversal of the blood-brain barrier (Kim, 2008). Type 1 fimbriae may also mediate adhesion of avian pathogenic *E. coli* to avian cells and tissues, and several attenuated APEC mutants were also reduced in their levels of production of type 1 fimbriae (Cortes et al., 2008; De Pace et al., 2010; Verma et al., 2015). Since a decrease in production of type 1 fimbriae in BEN2908Δ*treA* could be caused by accumulation of periplasmic trehalose, we investigated whether loss of endogenous trehalose synthesis in the Δ*otsBA* and Δ*treAotsBA* mutants would alter levels of type 1 fimbriae production. Yeast agglutination assays revealed that both of these mutants actually produced even less type 1 fimbriae than the *treA* mutant (**Figure 3A**). The effects of urea-mediated osmotic stress were not as adverse on the Δ*treA* mutant which showed greater resistance compared to WT, whereas the trehalose synthesis mutant Δ*otsBA* was more sensitive to urea-mediated stress. However, all trehalose mutants had decreased levels of production of type 1 fimbriae. Our hypothesis to explain this apparent paradox is that, in BEN2908, resistance to stress by urea would depend on the levels of periplasmic trehalose, but type 1 fimbriae production would rather be influenced by the levels of cytosolic trehalose. We hypothesize that the inability of BEN2908Δ*treA* to hydrolyze periplasmic trehalose would decrease the quantity of glucose entering the cytoplasm (through EIICB^Glu^), therefore lowering the rate of trehalose synthesis by OtsA-OtsB. The decrease in trehalose synthesis would not be as pronounced in Δ*treA* mutant compared to the Δ*otsBA* and Δ*treAotsBA* mutants, as reflected in the yeast agglutination assays. Li et al. (2012) previously reported that TreA activity was related to activities of OtsA-OtsB rather than directly to osmotic stress. If this interpretation proves correct, basal levels of cytosolic trehalose would be required for optimal production of type 1 fimbriae in ExPEC BEN2908; the mechanism of this regulation, however, remains to be elucidated. A diagram illustrating our hypothesis for the observed phenotypes of Δ*treA* and Δ*otsBA* mutants is shown in **Figure 7**.

Human urine has a high osmolality (average 500–600 mOsm/kg) and consists mainly of urea and some inorganic ions (mainly NaCl; (Ipe and Ulett, 2016)). Along with the osmoprotectants glycine-betaine and proline, trehalose is produced by *E. coli* to resist osmotic stress (Ruhal et al., 2013). In *E. coli*, trehalose as an osmoprotectant has been mainly investigated in response to stress by NaCl, whose effects on trehalose internal concentrations are a consequence of increased activities of OtsA and OtsB (Giaever et al., 1988) and increased expression of *otsA* and *otsB* (Withman et al., 2013). On the other hand, even though trehalose levels in bacterial cells growing in the presence of urea were not determined, increased expression of *otsA* and *otsB* was not detected in these conditions (Withman et al., 2013). Needless to say, however, the activity of an enzyme is subjected to regulation by other mechanisms (e.g., substrate concentrations, allosteric regulation) than only expression. Yet, according to our data, increased levels of periplasmic trehalose protected BEN2908 against urea (**Figure 1**; (Pavanelo et al., 2018).

Type 1 fimbriae expression is enhanced when UPEC is grown in urine (Snyder et al., 2004), and the component that would cause this enhancement is likely to be urea (Withman et al., 2013). As with *E. coli*, *Klebsiella pneumoniae* also has increased production of type 1 fimbriae when grown with 0.6 M urea (Lin et al., 2022). We also investigated whether trehalose synthesis by OtsA and OtsB were the mechanism by which urea stimulates type 1 fimbriae expression. However, in the Δ*otsBA* mutant which cannot produce endogenous trehalose, type 1 fimbriae production was also increased in the presence of 0.3 M urea (the average urea concentration in human urine) (**Figure 3B**). Thus, the mechanism by which urea acts on type 1 fimbriae production seems to be independent of trehalose levels in the bacterial cell.

Since production of type 1 fimbriae is energetically costly, it needs to be tightly regulated in *E. coli* (Blomfield and van der Woude, 2007). There are several mechanisms that control type 1 fimbriae, such as global regulators (H-NS, LRP, FNR) (Corcoran and Dorman, 2009; Barbieri et al., 2017), *ibeA* and *ibeT* genes and the phosphate (Pho) regulon (*pst*) (Cortes et al., 2008; Crépin et al., 2012b). More recently, several studies have shown that sugar metabolism plays a role in virulence of many gram-negative bacteria, such as *Ralstonia solanecearum* and *Burkholderia pseudomallei* (Vanaporn et al., 2017; MacIntyre et al., 2020). Based on that, it would not be surprising that some metabolites or osmolytes act as regulators of type 1 fimbriae. Here, we have shown that the removal of the genes responsible for metabolism of trehalose under high osmolality in BEN2908 resulted in decreased type 1 fimbriae production, as observed in reduced yeast agglutination, reduced capacity of adhesion to avian fibroblasts *in vitro* and reduced capacity of bladder colonization in a murine UTI model (**Figures 3**, **6**, **7**).

In summary, we generated BEN2908Δ*otsBA*, BEN2908Δ*treAotsBA* and the respective complemented mutants to better understand the relationship between trehalose metabolism and virulence of ExPEC. We have also shown that the loss of trehalose synthesis further impaired bladder colonization in a murine urinary tract infection model. Our results suggest that strain BEN2908 requires the osmoregulated enzymes of trehalose metabolism to optimally produce type 1 fimbriae, which is a key ExPEC virulence factor.

## 4 Materials and methods

### 4.1 Bacterial strains, plasmids, and growth conditions

All strains and plasmids are listed in **Table 1**. Strains and mutants were grown in Luria Bertani (LB) medium, LB with different amounts of NaCl or urea, or in M9 minimal medium containing 10 mM trehalose or 0.6% glycerol as the only carbon source. The following antibiotics were added when necessary: gentamicin 15 µg/µL, chloramphenicol 30 µg/µL, kanamycin 50 µg/µL or ampicillin 100 µg/µL. Before electroporation with pGpTn7-Gm plasmid, diaminopimelic acid (DAP)-negative MG617 strain was grown in the presence of DAP 50 µg/µL. Bacterial strains were stored in LB broth containing 20% glycerol at -80°C.

**Table 1:** Bacterial strains and plasmids used in this study.

Strain or plasmid	Characteristic	Resistance	Reference
BEN2908 (O2:H^+^: K1)	Wild type strain	Nalidixic acid	(Dho and Lafont, 1982)
BEN2908Δ*treA*	*treA*::Km/BEN2908Δ*treA*::scar (kanamycine sensitive)	Nalidixic acid	(Pavanelo et al., 2018)
BEN2908Δ*otsBA*	*otsBA*::Gm/BEN2908Δ*otsBA*::scar (gentamycin sensitive)	Nalidixic acid	This study
BEN2908Δ*treAotsBA*	*treA*::Km *otsBA*::Cm/ BEN2908Δ*treAotsBA::*scar (kanamycin and chloramphenicol sensitive)	Nalidixic acid	This study
BEN2908Δ*otsBA*/*otsBA* ^+^	BEN2908Δ*otsBA* + *treAotsBA, treA*::Km	Nalidixic acid, kanamycin	This study
BEN2908Δ*treAotsBA*/*treAotsBA* ^+^	Gm + *treAotsBA*	Nalidixic acid, gentamicin	This study
DH5αλpir	Vector strain	None	(Pósfai et al., 1997)
MGN-617	Donor strain. *thi thr leu tonA lacY glnV supE* Δ*asdA4 recA*::RP4 2-Tc::Mu [λpir] Km^r^	Kanamycin	(Dozois et al., 2000)
DM34	BEN2908Δ*fim*	Nalidixic acid	(Marc et al., 1998)
pKD46	λ-Red recombinase plasmid Ts replicon. Plasmid used for non polar mutation	Ampicilin	(Datsenko and Wanner, 2000)
pCP20	pCP20 FLP helper plasmid Ts replicon. Plasmid used to eliminate the gene cassette	Chloramphenicol, ampicillin	(Crépin et al., 2012a)
pGP-Tn7-Gm	pGP704::Tn7T-*Gm*. Plasmid used for complementation	Gentamicin and ampicillin	(Crépin et al., 2012a)
pSTNSK	pST76-K::*tnsABCD*. Plasmid used for complementation	Kanamycin	(Crépin et al., 2012a)
pkD3	Template plasmid for the amplification of the *cat* gene bordered by FRT sites	Chloramphenicol, ampicillin	(Datsenko and Wanner, 2000)
pkD4	Template plasmid for the amplification of the *kan* gene bordered by FRT sites	Kanamycin and ampicillin	(Datsenko and Wanner, 2000)

### 4.2 Construction of specific mutants and complemented strains

All mutants were generated by the lambda-Red recombinase technique (Datsenko and Wanner, 2000). Genes were disrupted by inserting a resistance gene cassette, using pDK3 or pKD4 plasmids as templates for chloramphenicol and kanamycin resistance, respectively. To eliminate any potential polar effects, the cassettes, flanked by FRT sites, were eliminated using the Flp resolvase expressed from plasmid pCP20 (Crépin et al., 2012a).

Chromosomal complementation was achieved by insertion of genes at the *att*Tn7 site on the bacterial chromosome as previously described (Crépin et al., 2012a). Briefly, the *otsBA* operon and its promoter region from *E. coli* BEN2908 were cloned into pGpTn7-Gm in *E. coli* strain DH5α *pir*, generating pGpTn7-Gm-*otsBA*. The *treA* gene and its promoter were then amplified from genomic DNA of BEN2908 and cloned into the pGpTn7-Gm-*otsBA* plasmid in *E. coli* strain DH5α *pir*, generating pGpTn7-Gm-*treAotsBA*. The Δ*otsBA* and Δ*treAotsBA* strains were electroporated with plasmid pSTNSK and grown at 30 °C. The generated Δ*otsBA* + pSTNSK and Δ*treAotsBA* + pSTNSK strains were mated with donor strain MGN-617 pGpTn7Gm+*treAotsAB*, generating the complemented strains Δ*otsBA*/*treAotsBA* ^+^ and Δ*treAotsBA*/*treAotsBA* ^+^. The complementations were achieved through single-copy integration in trans at the attTn7 site, upstream the *glmS* gene. Since the Δ*otsBA*/*treAotsBA* ^+^ strain contained two copies of *treA*, the *treA* gene and Gm resistance cassette at the *att*Tn7 site were replaced with a Km cassette, generating the complemented Δ*otsBA*/*otsBA^+^* strain (Datsenko and Wanner, 2000). All primers used are listed in **Table 2**.

**Table 2:** Primers used in this study.

Primers	Sequence	Function	Reference
CMD 2072 _screening_otsAB_F	TGCAAATGGCGACCCCCGTC	Confirm CmR cassete removal from Δ*otsBA*	This study
CMD_2073_ screening_otsAB_R	AGCTGCGCCGATGCTTGAAGA	Confirm CmR cassete removal from Δ*otsBA*	This study
CMD_2005_ screening_treAKO_F	ACCGTTCGGATGGCATCATT	Confirm KmR cassete removal from Δ*treotsBA*	Pavanelo et al., 2018
CMD_2018_treAcompl_R	AGAAACTAGTGGCATAGACCGTAGAATGGGGG	Confirm KmR cassete removal from Δ*treotsBA*	This study
CMD_2447_ otsBA_compl_R	GGGCTGCAGGAATTCCTCGAGCCCGTTGACAAGACGTTTATTGC	Complementation confirmation of Δ*otsBA* and Δ*treAotsBA*	This study
CMD_ 1073_pstS_tn7_R	AGATCAGTTTGGTGTACGCCAGGT	Complementation confirmation of Δ*otsBA* and Δ*treAotsBA*	Crépin et al., 2012a
CMD_26_glmS	GATCTTCTACACCGTTCCGC	*treA* knockout from *ΔotsBA + treAotsBA*	Crépin et al., 2012a
CMD_267_Km cassete	CGGTGCCCTGAATGAACTGC	*treA* knockout from *ΔotsBA + treAotsBA*	Crépin et al., 2012a

### 4.3 Yeast agglutination assay

The strains were inoculated from an overnight pre-inoculum in LB medium and grown in LB broth at 37° C under shaking (240 rpm) until mid-log phase (OD_600_ = 0.6). A suspension of 2 x 10^9^ bacterial cells was serially diluted 1:2 in phosphate buffered saline (PBS) in a microtiter plate. A 1.5% suspension of commercially available yeast was added to each well. After 30 minutes incubation on ice, agglutination was visually monitored and the agglutination titer was determined as the most diluted well wherein agglutination was observed. In order to verify the influence of urea on production of type 1 fimbriae, yeast agglutination assays were performed as above, except that strains were grown in LB broth without NaCl in the absence (control) or presence of 0.3 M urea.

### 4.4 Growth in conditions of osmotic stress

Strains were tested for the capacity to grow under conditions of osmotic stress caused by NaCl or urea. Strains were inoculated 1:100 from an overnight pre-inoculum in LB broth and grown until mid-log phase (OD_600_ = 0.6) under shaking (240 rpm) at 37° C. They were serially diluted and plated on LB agar without NaCl or on LB agar plates with 0.3 M NaCl, 0.6 M NaCl, 0.3 M urea or 0.6 M urea. After incubation at 37 °C for 24 h, colonies were counted and growth under each condition was compared to growth on LB agar without NaCl.

### 4.5 Growth on minimal medium with trehalose as the only carbon source

To verify if the mutants retained their ability to use trehalose as a sole carbon source, BEN2908 and mutants were grown in M9 medium plates (60 g/L Na_2_HPO_4_, 30 g/L KH_2_PO_4_, 5 g/L NaCl, 10 g/L NH_4_Cl; 0.5 mL of MgSO_4_.7H_2_O 1 M, 0.5 mL of CaCl_2_ 0.1 M and 1 mL of thiamine 0.5 M; 1.5% agar) with 0.6% glycerol or 10 mM trehalose as the only carbon source. Strains were inoculated 1:100 from an overnight preinoculum in LB broth and grown until mid-log phase (OD_600_ = 0.6) under shaking at 37 °C. All of them were serially diluted and plated on M9 agar with 0.6% glycerol or 10 mM trehalose. After incubation at 37 °C for 48 h, colonies were counted and growth on trehalose was compared to growth on glycerol.

### 4.6 Swimming motility assay

Swimming motility was done as previously described (Verma et al., 2015). An overnight culture of each strain was diluted 1:100 in LB broth and grown at 37° C under shaking until mid-log phase (OD_600_ = 0.6). Cultures of each strain were stabbed into the surface of LB 0.3% (soft) agar using an inoculating needle and care was taken not to touch the bottom of the plate during inoculation to ensure only swimming motility was assessed. The plates were incubated face up at 37° C for 16 hours. Three independent experiments were performed for each strain.

### 4.7 Cell adhesion and invasion assays

Bacteria-eukaryotic cell association assays were performed with CEC-32 avian fibroblasts as described previously (Pavanelo et al., 2018). Briefly, 5 x 10^4^ cells/well were distributed in 96-well plates in DMEM high glucose with 10% fetal bovine serum. On the following day, cultures were washed once with PBS to remove dead cells and returned to the culture medium. Bacterial strains were inoculated from an overnight pre-inoculum in LB medium and grown at 37° C under shaking (240 rpm) until mid-log phase (OD_600_ = 0.6). Cells were infected at a multiplicity of infection (MOI) of 20 bacteria per cell. After 1 h of incubation at 37°C and 5% of CO_2_, the medium was removed, and the cells were washed 3 times with PBS. For the adhesion assays, cells were then lysed with Triton X-100 1% (v/v) in PBS for 5 min. Lysates were serially diluted and plated on LB agar for CFU counting. For the invasion assays, cells were further incubated in culture medium supplemented with 50 µg/ml gentamicin for 3 h, after which they were lysed, and lysates diluted and plated.

### 4.8 Experimental urinary tract infection

Experimental urinary tract infections were performed as previously described (Hagberg et al., 1983). Five-week-old-female CBA/J mice were infected with 20 µL (2 x 10^9^ CFU) using a catheter to insert the bacterial suspension through the urethra. At 48 hours post-infection, mice were euthanized and the bladder and both kidneys were collected, homogenized in PBS, serially diluted and plated onto MacConkey-agar; plates were incubated at 37° C for 16 hours for colony counting.

### 4.9 Statistical analysis

The Mann-Whitney test was used to compare the samples by pairs, and Kruskal Wallis was used to compare groups, since distribution of results was nonparametric. Tests were performed using GraphPad Prism version 6.00 for Windows, GraphPad Software, La Jolla California USA, www.graphpad.com.

### 4.10 Animal ethics statement

The protocol for mice urinary tract infection was approved by the animal ethics evaluation committee – *Comité Institutionel de Protection des Animaux* (CIPA No. 1608–02) of the INRS-Centre Armand-Frappier.

## Data availability statement

The original contributions presented in the study are included in the article/supplementary material. Further inquiries can be directed to the corresponding author.

## Ethics statement

The animal study was approved by Comité Institutionel de Protection des Animaux (CIPA No. 1608–02) of the INRS-Centre Armand-Frappier. The study was conducted in accordance with the local legislation and institutional requirements.

## Author contributions

VK: Data curation, Formal analysis, Investigation, Methodology, Writing – original draft, Writing – review & editing. DP: Conceptualization, Formal analysis, Methodology, Supervision, Writing – review & editing, Writing – original draft. SH: Data curation, Investigation, Methodology, Writing – review & editing. SD: Investigation, Methodology, Writing – review & editing. PP: Investigation, Methodology, Writing – review & editing. SI-J: Investigation, Methodology, Writing – review & editing. CD: Conceptualization, Formal analysis, Funding acquisition, Project administration, Supervision, Writing – review & editing. FH: Conceptualization, Formal analysis, Funding acquisition, Investigation, Methodology, Project administration, Supervision, Writing – review & editing, Writing – original draft.
